# Hemodynamic changes and mid-term results of surgical correction of de novo aortic valve insufficiency after left ventricular assist device implantation

**DOI:** 10.1007/s10047-025-01516-9

**Published:** 2025-06-28

**Authors:** Takashi Murakami, Yusuke Misumi, Daisuke Yoshioka, Takuji Kawamura, Ai Kawamura, Shin Yajima, Shunsuke Saito, Takashi Yamauchi, Shigeru Miyagawa

**Affiliations:** Department of Cardiovascular Surgery, Osaka University Graduate School of Medicine, 2-2-E1, Yamadaoka, Suita, Osaka 565-0871 Japan

**Keywords:** Ventricular assisted device, Aortic insufficiency

## Abstract

Severe aortic insufficiency (AI) is a common complication associated with prolonged continuous-flow left ventricular assist device (CF-LVAD) therapy. This study aimed to investigate the clinical outcomes after surgical correction of de novo AI after LVAD implantation. A total of 190 patients underwent CF-LVAD implantation between January 2013 and June 2022. Of these, 24 had trivial or no AI before LVAD implantation and developed moderate or greater de novo AI after LVAD implantation. Patients who underwent aortic valve surgery before or concomitant with LVAD surgery were excluded. Among the 24 patients, surgeries were indicated for medically refractory de novo AI in 11 patients, who were included. The primary outcome was postoperative improvement in hemodynamics as assessed by right heart catheter examination, and the secondary endpoints were 3-year survival and freedom from death and/or heart failure readmission rates. The correction of de novo AI was accomplished with aortic valve closure using a bovine pericardial patch in 10 patients and prosthetic valve replacement in one patient. Significant differences (all *p* < 0.01) in pre- vs. post-surgery pulmonary artery wedge pressure, cardiac index, and mixed venous blood oxygen saturation were found. The mean follow-up period after LVAD implantation was 1413 days, and the 3-year survival rate was 90.9%. Three-year freedom from postoperative moderate or greater AI rate and freedom from heart failure readmission rate were both 90.9%. Postoperative hemodynamic status and survival outcomes are favorable in patients who underwent surgical aortic valve repair de novo AI after LVAD implantation.

## Introduction

Left ventricular assist devices (LVADs) have become an effective treatment option for severe heart failure [1] and recently described continuous flow left ventricular assist devices (CF-LVADs) improve survival and quality of life in patients with end-stage heart failure [2–4]. As survival outcomes in patients undergoing LVAD therapy have become more favorable, the long-term use of LVADs puts patients at high risk of hemorrhage, cerebrovascular disease, pump thrombosis, infection, and the development of aortic insufficiency (AI), with an increasing prevalence of associated adverse events [5]. Due to the improved performance of LVADs, some complications such as thrombosis are less common, while others still occur such as bleeding complications and right heart failure. Among them, the most significant long-term complication is AI. Up to 30% of patients with CF-LVADs develop moderate AI or worse [6–9] and once de novo AI occurs after LVAD implantation, its severity progresses [7]. AI also forms a circulatory loop between the left ventricle and the aorta, causing increased left-sided filling pressures, worsening heart failure symptoms, and impaired exercise tolerance and end-organ perfusion [10, 11]. Conservative treatment with diuretics for volume load reduction and vasodilators for afterload reduction is used to overcome the detrimental effects of symptomatic AI [12–14]. Although increased pump speed can temporarily compensate for the reduction in effective cardiac flow, surgical management strategies when conservative treatment fails include Park’s stitch, modified Park’s stitch, complete closure of the ventricular-aortic junction with an acicular patch or surgical bioprosthetic valve [15, 16]. However, despite reports of high operative mortality rates for surgical intervention for aortic valve in patients with LVAD [17], few studies have investigated the hemodynamic status before and after surgical intervention for severe AI. Therefore, this study aimed to evaluate hemodynamic status and survival after aortic valve repair in patients with symptomatic aortic regurgitation undergoing LVAD therapy.

## Methods

### Patients

**Fig. 1:**
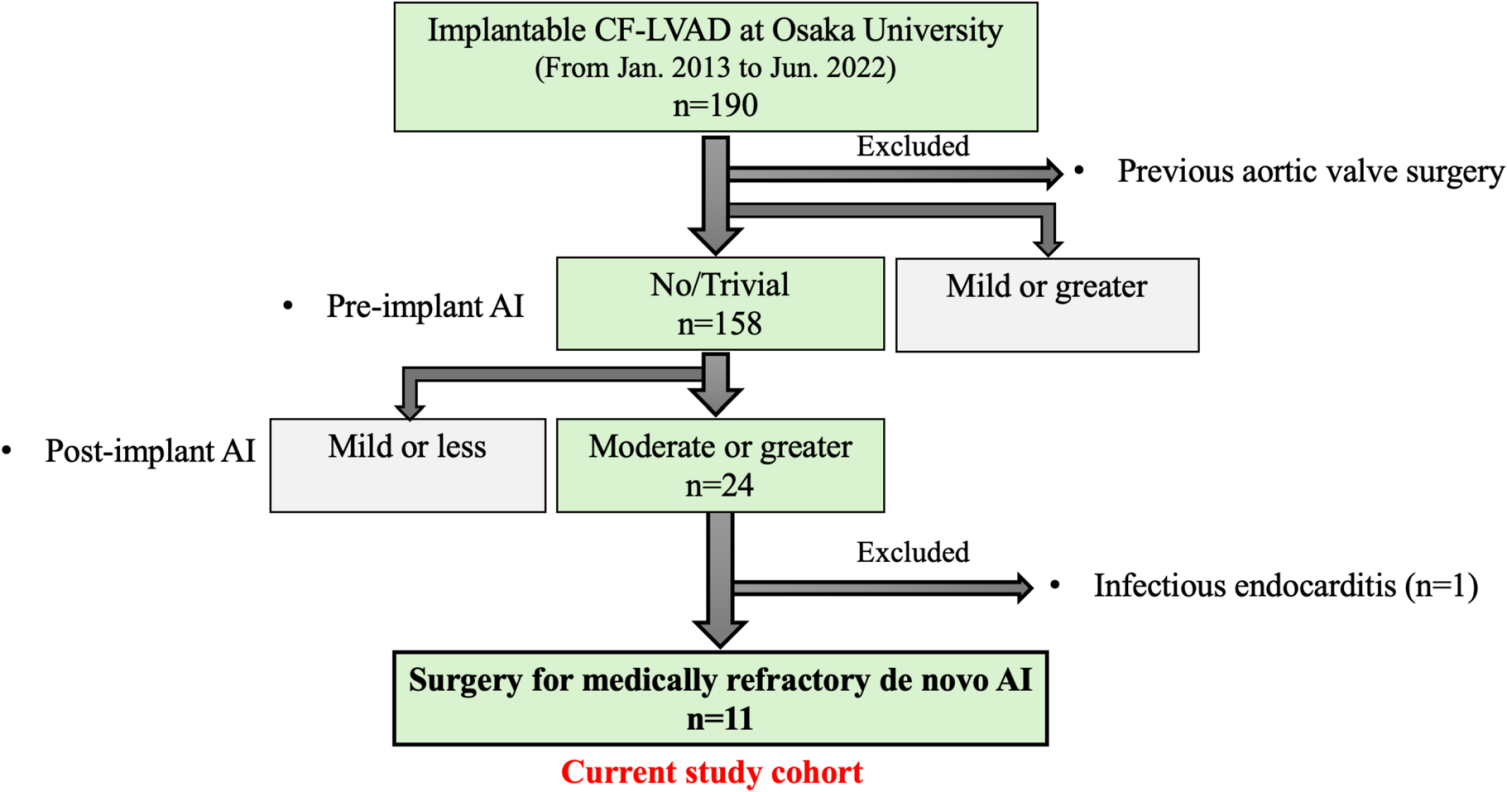
Participant selection flow chart. Between January 2013 and June 2022, 190 patients underwent continuous-flow LVAD implantation. Of these, 24 patients had trivial or low AI before LVAD surgery and developed moderate or high de novo AI after LVAD implantation. The patients who underwent aortic valve surgery before or concomitant with LVAD surgery were excluded. Among the 24 patients, surgery was indicated for medically refractory de novo AI in 11 who were included in the current study.\

Among the 190 patients with implantable LVADs at Osaka University Hospital between January 2013 and June 2022, 11 (5.8%) with a preoperative AI of none or trivial grade that progressed to more than or equal to moderate postoperatively and required surgical intervention were included. Thirteen patients with postoperative progression above moderate who did not require surgical interventions and one patient with aortic valve endocarditis were excluded (Fig. 1). The changes in hemodynamics and symptoms before and after aortic valve intervention were retrospectively analyzed. Survival and freedom from heart failure hospitalizations were also retrospectively analyzed. The median follow-up period after surgical correction of de novo AI was 1413 days (range 1289–1557 days). The indications for surgical treatment of AI were assessed by a multidisciplinary team of cardiac surgeons, interventional cardiologists, and heart failure cardiologists. The cardiology team determined the need for surgical intervention for aortic valve in treatment refractory cases, such as failure to improve heart failure symptoms or poor right heart catheterization results despite maximum medical treatment, including the use of diuretics and vasodilators, and LVAD rotation adjustments. The presence or absence of AI and its severity were determined before surgical intervention and after surgical intervention at follow-up periods of 1, 3, 6, 12, 24, and 36 months.

### Surgical procedure for aortic valve

After establishing a cardiopulmonary bypass via median re-sternotomy, the outflow graft of the LVAD was clamped, the LVAD drive was stopped, and the outflow graft was completely dissected. A catheter for the antegrade cardioplegia was inserted into the distal side of the outflow graft and a left ventricular vent tube was inserted into the proximal side of the outflow graft. The aortic root was thoroughly dissected. After the ascending aorta was clamped, cold-blood antegrade and succeeding retrograde cardioplegia were administered for rapid cardiac arrest. After removing the aortic valve, the ventricular-aortic junction diameter was measured. A two-layer bovine round pericardial patch was cut as a closure patch and secured in the supra-annular position with 15 pairs of mattress 2–0 polyester braded sutures with a pledget. After the procedure, the patient was weaned from the cardiopulmonary bypass and the LVAD drive was restarted. Finally, transesophageal echocardiography was used to confirm the absence of AI.

### Statistical methods

All data analyses were performed using JMP software (version 17.0; SAS Institute Inc.). Data were expressed as quartile means or mean ± standard deviation or median and range for continuous variables and numerical values (percentages) for categorical variables. Continuous variables were compared using the Mann–Whitney U test. Survival and readmission avoidance rates were analyzed using the Kaplan–Meier method.

## Results

### Patients’ characteristics

The median (range) age at the time of surgical intervention was 51 (41–60) years, and 72.7% of the patients were male (Table 1). None of the patients had significant AI at the time of LVAD implantation. The mean follow-up period after LVAD implantation was 1413 days, and the mean duration to the first moderate or greater AI development after LVAD implantation was 13 months. The mean time to the first hospitalization for heart failure after LVAD implantation was 13 months. The average time from LVAD implantation to the failure of conservative treatments for heart failure requiring aortic valve closure or replacement was 21 months (Table 2).

**Table 1:** Patients’ characteristics before left ventricular assist device implantation

Patients’ characteristics	
Age, median (Q1–Q3)	50 (40–59)
Male, *n* (%)	8 (73%)
BMI (kg/m^2^), median (Q1–Q3)	21.2 (18–23.8)
BSA (m^2^), median (Q1–Q3)	1.65 (1.42–1.84)
Candidate to DT, *n* (%)	2 (18%)
Etiology	
DCM, *n* (%)	5 (45%)
ICM, *n* (%)	3 (27%)
DHCM, *n* (%)	2 (18%)
Postpartum cardiomyopathy	1 (9%)
Coexisting diseases	
Previous cardiac surgery, *n* (%)	1 (9%)
Hypertension, *n* (%)	1 (9%)
Dyslipidemia, *n* (%)	2 (18%)
Diabetes mellitus, *n* (%)	1 (9%)
COPD, *n* (%)	0 (0%)
CVA, *n* (%)	0 (0%)
Peripheral vascular disease, *n* (%)	0 (0%)
Preoperative condition	
INTERMACS profile, median (Q1–Q3)	2 (2–3)
IABP, *n* (%)	0 (0%)
PCPS, *n* (%)	0 (0%)
IMPELLA, *n* (%)	1 (9%)
Preoperative echocardiography	
LVDd (mm), median (Q1–Q3)	68 (56–74)
LVDs (mm), median (Q1–Q3)	62 (46–67)
LVEF (%), median (Q1–Q3)	23 (14–33)
No AI, *n* (%)	5 (45%)
Trivial AI, *n* (%)	6 (55%)
Significant MR, *n* (%)	5 (45%)
Significant TR, *n* (%)	4 (36%)
Preoperative RHC	
Mean PAP (mmHg), median (Q1–Q3)	28 (21–36)
PCWP (mmHg), median (Q1–Q3)	18 (15–23)
CI (L/min/m^2^), median (Q1–Q3)	2.17 (1.79–2.42)
Preoperative blood tests	
Total protein (g/dL), median (Q1–Q3)	7.3 (6.8–7.5)
Albumin (g/dL), median (Q1–Q3)	4.1 (3.8–4.3)
Hemoglobin (g/dL), median (Q1–Q3)	13.5 (11.8–15.2)
AST (IU/L), median (Q1–Q3)	25 (23–36)
ALT (IU/L), median (Q1–Q3)	21 (15–37)
Total bilirubin (g/dL), median (Q1–Q3)	1.5 (1.1–2.1)
Creatinine (mg/dL), median (Q1–Q3)	1.14 (0.91–1.3)
BNP (pg/mL), median (Q1–Q3)	197 (81–382)

*AI* Aortic Insufficiency, *ALT* Alanine Aminotransferase, *AST* Aspartate Aminotransferase, *BMI* Body Mass Index, *BNP* B-Type Natriuretic Peptide, *BSA* Body Surface Area, *CI* Cardiac Index, *COPD* Chronic Obstructive Pulmonary Disease, *CVA* Cerebrovascular Accident, *DCM* Dilated Cardiomyopathy, *DHCM* Dilated-phase Hypertrophic Cardiomyopathy, *IABP* Intra-Aortic Balloon Pump, *ICM* Ischemic Cardiomyopathy, *LVD* Left Ventricular Dysfunction, *LVDd* Left Ventricular End-Diastolic Dimension, *LVEF* Left Ventricular Ejection Fraction, *MR* Mitral Regurgitation, *PAP* Pulmonary Artery Pressure, *PCPS* Percutaneous Cardiopulmonary Support, *PCWP* Pulmonary Capillary Wedge Pressure, *RHC* Right Heart Catheterization

**Table 2:** Patients’ background

Patients’ background	
Follow-up duration after LVAD implantation, (day) median (Q1–Q3)	1413 (1289–1557)
Number of heart failure readmission, n median (Q1–Q3)	1 (1–2)
Duration from LVAD implantation to:	
De novo AI development, (month) median (Q1–Q3)	13 (7–18)
First heart failure readmission, (month) median (Q1–Q3)	13 (7–18)
Surgery for de novo AI, (month) median (Q1–Q3)	21 (15–37)

*AI* Aortic Insufficiency, *LVAD* Left Ventricular Assist Device

### *Early clinical outcomes after surgery for *de novo* AI*

Ten patients underwent aortic valve patch closure, and in one patient, biological aortic valve replacement was performed because of the possibility of weaning from the LVAD and the desire to become pregnant in the future. Data for surgical procedure type, concomitant procedures, and postoperative complications are shown in Table 3. All patients survived the surgery and no 30-day postoperative deaths were reported. The median ICU stay after the surgical intervention was 9 (7–35) days. All patients were discharged safely. Post-operative hemostasis was required in 3 of the 11 patients due to hemorrhage. Postoperative cerebrovascular accidents or renal failure were not observed (Table 3).

**Table 3:** Surgical data

Surgical data	
Aortic valve procedure	
Biological valve replacement, *n* (%)	1 (9%)
Bovine patch closure, *n* (%)	10 (91%)
Device	
Heart Mate II	6 (55%)
Jarvik2000	2 (18%)
EVAHEART	1 (9%)
EVAHEART II	1 (9%)
HVAD	1 (9%)
Concomitant procedure	
Tricuspid annuloplasty	5 (45.4%)
LVAD exchange	3 (27.3%)
Temporary RVAD	3 (27.3%)
Procedure duration (min) (mean ± SD)	486 ± 108
CPB time (min) (mean ± SD)	196 ± 68
Cross clamp time (min) (mean ± SD)	81 ± 30
30-day mortality, *n* (%)	0
CVA, *n* (%)	0
New renal failure, *n* (%)	0
New dialysis, *n* (%)	0
Deep sternal infection, *n* (%)	1 (9%)
Re-exploration for hemorrhage, *n* (%)	3 (27.3%)
ICU stay (days), median (Q1–Q3)	9 (7–35)

*CPB* Cardiopulmonary Bypass, *CVA* Cerebrovascular Accident, *ICU* Intensive Care Unit, *LVAD* Left Ventricular Assist Device; *RVAD* Right Ventricular Assist Device, *SD* Standard Deviation

### *Hemodynamic improvement after surgical correction of *de novo* AI*

**Fig. 2:**
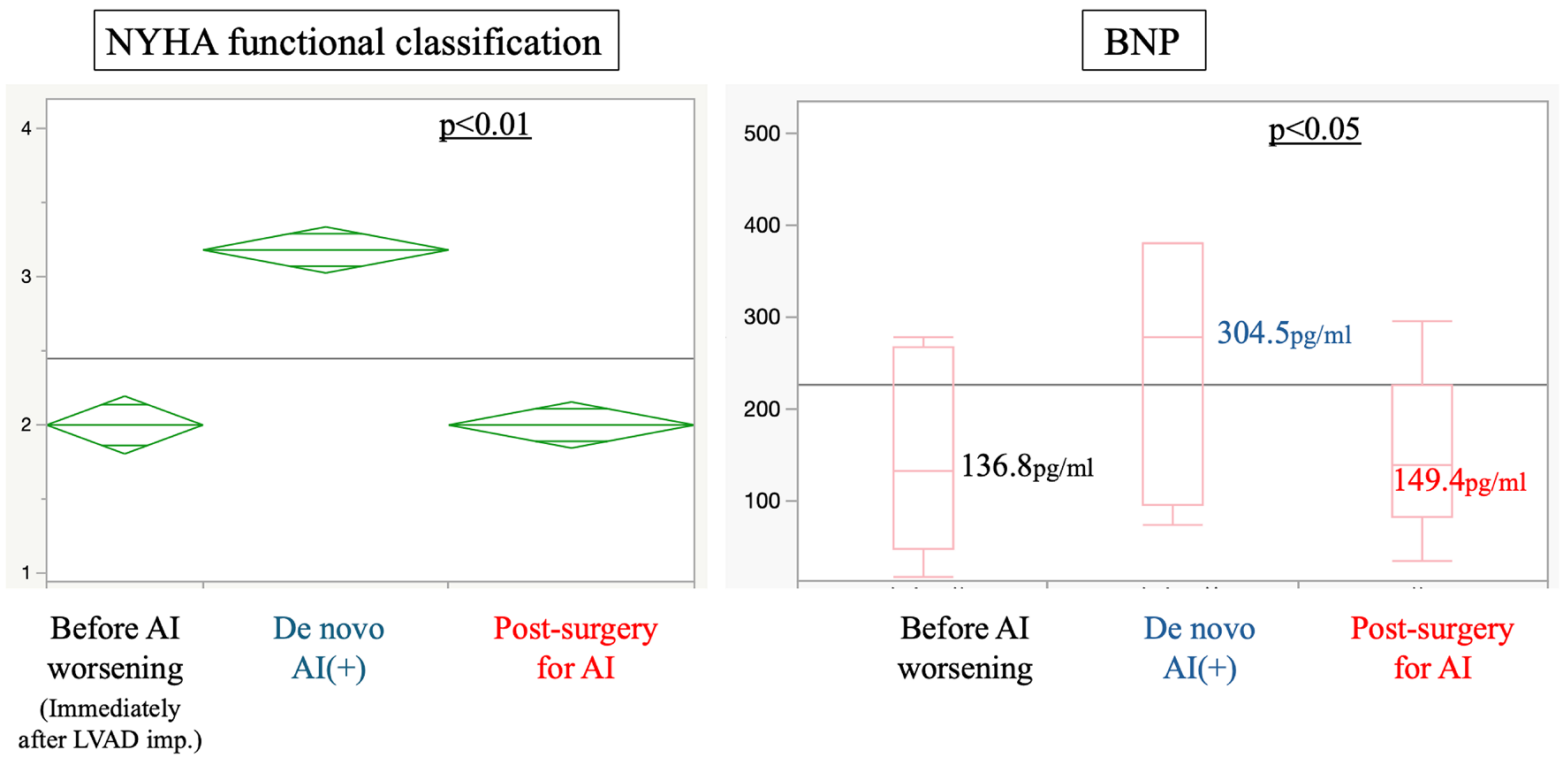
Changes in NYHA functional classification and BNP level. New York Heart Association functional classification and brain natriuretic peptide level improved compared with the preoperative baseline

**Fig. 3:**
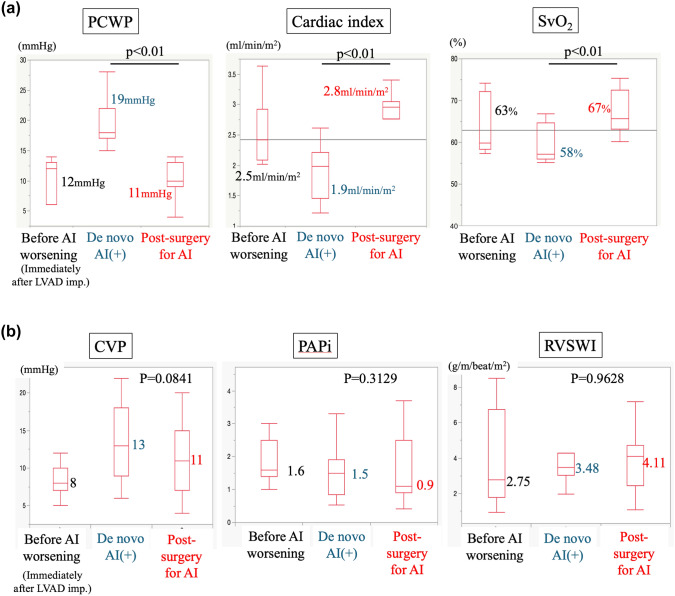
Hemodynamic changes. Hemodynamic status as measured by pulmonary capillary wedge pressure, cardiac index, and mixed venous oxygen saturation (**A**), and central venous pressure, pulmonary artery pulsatility index, and right ventricular stroke work index (**B**) is represented in the immediate post-LVAD state, in the state of heart failure due to de novo AI, and after surgical repair of the aortic valve. All indices significantly improved after aortic valve repair

In 10 out of the 11 patients, AI completely resolved after aortic valve closure or replacement, and at 18 months, one patient developed severe AI. All patients listed as bridge-to-transplant were still receiving LVAD therapy 12 months after surgical intervention. Significant improvement was shown in the New York Heart Association functional classification (*p* < 0.01), and in brain natriuretic peptide (BNP) level, from 278.1 [96–381.9] pg/ml to 133 [47.7–267.1] pg/ml (p < 0.05) (Fig. 2). Hemodynamics were significantly altered at each time point, i.e., immediately after LVAD implantation, at heart failure after development of de novo AI, and after intervention for aortic valve (Fig. 3A). Hemodynamic indices significantly improved after aortic valve closure or replacement (all* p* < 0.01). Pulmonary capillary wedge pressure increased from 12 to 19 mmHg and decreased to 11 mmHg after intervention for aortic valve. The cardiac index decreased from 2.5 to 1.9 and improved to 2.8. Mixed venous oxygen saturation was 63% and decreased to 58% with worsening AI but improved to 67%. The timing of the deterioration of these parameters was prior to surgical intervention on the aortic valve, measured at the time of hospitalization for heart failure due to AI or during preoperative right heart catheterization.

Similarly, parameters related to right heart function, such as central venous pressure (CVP), pulmonary artery pulsatility index (PAPi), and right ventricular stroke work index (RVSWI), were also analyzed, but no significant differences were detected (Fig. 3B).

### *Mid-term results after surgery for *de novo* AI*

**Fig. 4:**
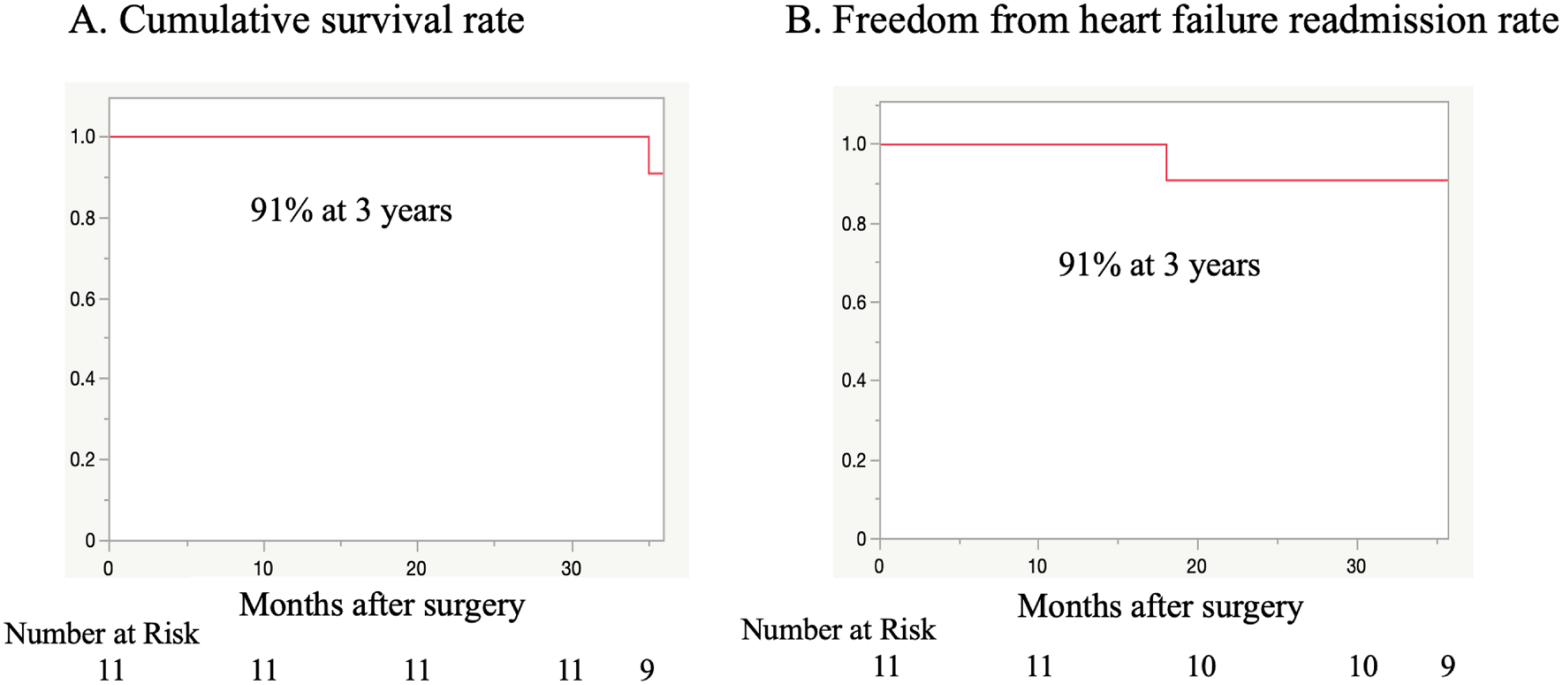
Survival and readmission rate. The 3-year survival rate was 91% (**A**). Freedom from heart failure readmission rates were 91% at 3 years (**B**)

Figure 4 presents the cumulative survival and readmission rates. The 3-year survival rate was 91% (Fig. 4a), and the rate of freedom from postoperative moderate or worse AI and freedom from rehospitalization for heart failure was 91% at 3 years (Fig. 4b). Only one patient was hospitalized for heart failure because of recurred AI after aortic valve patch closure. The patient underwent aortic valve closure again but ultimately died from a refractory LVAD pump infection. The patch was excessively folded at the annulus on the right coronary cusp side, from which the AI was thought to have remained.

Of the 10 surviving patients, five have undergone heart transplantation after a waiting period, and the remaining five were still undergoing LVAD therapy.

## Discussion

In the present study, surgical correction for de novo AI was indicated for patients with more advanced heart failure than in previous studies. Grinstein et al. [18] reported that right heart catheterization results varied widely according to the severity of AI in patients with LVADs, with pulmonary artery wedge pressures of 10.2 mmHg in the no-AI group and 15 mmHg in the moderate or higher group. In the current study, the patients showed pulmonary artery wedge pressures of 19 mmHg after development of de novo AI, which indicates apparently advanced heart failure compared with previous reports. However, surgical correction of de novo AI was safely done in all patients and significant improvement in hemodynamic parameters such as pulmonary capillary wedge pressure was confirmed.

When prosthetic valve replacement is performed in non-LVAD patients with chronic AI, left ventricular reverse remodeling and hemodynamic improvement often take up to 6 months. [19] In chronic AI, the left ventricle is capacitively loaded for a reasonable time before surgery is indicated and remodeling progresses. In contrast, the patients with LVAD have end-stage heart failure as a result of cardiomyopathy or myocardial infarction, and their cardiac function is severely impaired as a result of progressive cardiac remodeling. De novo AI after LVAD implantation cannot compensate for the increased volume load caused by the blood ejected by the LVAD returning into the left ventricle and this leads to heart failure. Staving off this “re-circulation” restores the efficiency of the LVAD and results in an immediate hemodynamic improvement. In other words, in terms of left heart parameters (PCWP, CI and SvO2), physical improvement of AI may have improved early postoperative hemodynamics by allowing enough unloading of LV and enough output to systemic circulation in patients dependent on LVAD. In addition, based on the values of right heart parameters (CVP, PAPi and RVSWI), early surgical intervention for aortic valve before right heart failure progressed may have led to a good outcome.

As for a technique of surgical correction of AI, Park’s stitch is a simple and widely used especially for de novo AI management in LVAD patients [15] but recurrence after long-term follow-up has been reported [20, 21]. To achieve complete control of AI, we used patch closure of the valve despite it requires a slightly longer cardiac arrest time. [22]

Overall, the postoperative survival rates after surgical intervention for AI in patients with LVAD in this study seems better compared with those reported in previous studies. Adamson et al. [16] performed aortic valve closure in 28 patients and reported a 1-year survival rate of 78% and a 3-year survival rate of 53%. Rao et al. [17] performed surgical intervention for aortic valve in seven patients, with a 1-year survival rate of 71.4%. Yehya et al. [23] performed transcatheter aortic valve implantation in nine patients and reported a 1-year survival rate of 56%, while Phan et al. [24] performed transcatheter aortic valve implantation or percutaneous occlusion device therapy on 29 patients and reported an in-hospital mortality rate of 31%. Transcatheter aortic valve implantation and percutaneous occlusion device therapy may be advantageous in terms of surgical invasiveness. However, off-label use and serious complications have been reported; therefore, the percutaneous treatment of de novo AI requires careful consideration. Aortic valve closure or replacement via re-sternotomy may be a reasonable therapeutic approach when AI in patients with LVADs can no longer be managed conservatively.

Our study has several limitations. As a single-center study, the sample size was small. However, no previous studies on de novo AI after LVAD implantation requiring surgical intervention involving large samples have been reported. Few reports of hemodynamic improvement after aortic valve repair via re-sternotomy in patients with severe de novo AI after LVAD implantation or of outcomes in such patients are available. The novelty of this study lies in the analysis of hemodynamics before and after intervention for aortic valve in this patient population. Our report provides valuable information on these issues despite the small sample size. Additionally, quantitative assessment of AI severity after LVAD placement is difficult, and no consensus method is currently available. Finally, long-term follow-up data to assess long-term outcomes were lacking.

In conclusion, our findings suggest that postoperative hemodynamic status and survival rates are favorable in patients who underwent aortic valve closure or replacement for de novo AI after LVAD implantation.

## Data Availability

Data sharing is not applicable to this article as no new data were created or analyzed in this study.

## Abbreviations

**AI**: Aortic insufficiency
**CF-LVAD**: Continuous-flow left ventricular assist device
**LVAD**: Left ventricular assist device
